# Metal‐Organic Framework‐Based Tribovoltaic Textile for Human Body Signal Monitoring

**DOI:** 10.1002/advs.202414086

**Published:** 2025-02-05

**Authors:** Yuanlong Li, Yinghong Wu, Alexander V Shokurov, Carlo Menon

**Affiliations:** ^1^ Biomedical and Mobile Health Technology Laboratory Department of Health Sciences and Technology ETH Zurich Lengghalde 5 Zürich 8008 Switzerland; ^2^ National Engineering Research Center of Green Recycling for Strategic Metal Resources Institute of Process Engineering Chinese Academy of Sciences Beijing 100190 China

**Keywords:** human motion monitoring, metal‐organic framework, Schottky contact, smart textile, tribovoltaic nanogenerator

## Abstract

The pursuit of sustainable and portable direct current (DC) energy suppliers has ignited considerable interest in tribovoltaic nanogenerators (TVNGs), devices that harvest mechanical energy from the surrounding environment. However, the predominant focus in TVNG research has centered on rigid and silicon‐based semiconductors that lack flexibility and are thus ill‐suited for integration into common fabrics. Herein, a fully‐textile TVNG with a simple design is introduced that enables the real‐time monitoring of human physiological signals. The utilization of copper‐benzenehexathiol (Cu‐BHT), a conductive 2D metal‐organic framework is proposed as a p‐type semiconductor grown on fabric surfaces. The developed tribovoltaic textile (TVT) consists of Cu‐BHT‐modified cotton and metallic aluminum textile producing pure DC output due to self‐rectification. With excellent flexibility and stability, Cu‐BHT TVT is seamlessly integrated into textile‐based accessories for continuous monitoring of human motion and respiration.

## Graphical abstract

A metal‐organic framework‐based tribovoltaic textile (TVT) is developed using copper‐benzenehexathiol (Cu‐BHT) as a novel p‐type semiconductor. The Cu‐BHT TVT consistently produces pure DC output due to self‐rectification and exhibits excellent flexibility and stability. When integrated into garments, the full‐textile device can monitor human body signals via harvesting motion energy, demonstrating its great potential in sports and healthcare.

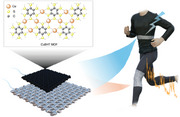

## 1 Introduction

The rapid development of wearable electronics has brought significant changes to our daily lives, particularly in areas such as health monitoring,^[^ ^1^, ^2^, ^3^, ^4^, ^5^ ^]^ communication, and application implementation.^[^ ^6^ ^]^ Electronic textiles (e‐textiles) represent one of the most promising technologies in wearables due to their essential role in everyday wear, along with outstanding flexibility, breathability, and comfort.^[^ ^7^, ^8^ ^]^ Despite remarkable advancements, e‐textiles still encounter the same challenge as traditional wearables: their power supplies, like batteries and supercapacitors, are often limited by bulkiness, the need for encapsulation, and unsafety.^[^ ^9^ ^]^ In recent years, considerable efforts have been made to develop new energy harvesting technologies. Notably, triboelectric textile (textile‐based triboelectric nanogenerator, t‐TENG),^[^ ^10^, ^11^, ^12^ ^]^ capable of harvesting electricity from mechanical energy, used both for sensing and powering of other devices, seems particularly perspective owing to broad material selection, simple fabrication, high output voltage, etc.^[^ ^13^ ^]^

The last decade has witnessed impressive progress of t‐TENGs in materials innovation, device configuration, and application exploration. However, conventional t‐TENGs often encounter two main challenges. First, similar to non‐textile TENGs, t‐TENGs typically exhibit high device impedance and produce alternating current (AC) output.^[^ ^12^ ^]^ This necessitates the use of rectifiers and often results in impedance mismatches with electronic components that they are intended to power, limiting the practical applications. While some solutions to alleviate this problem exist, e.g. the direct current (DC) TENGs via air discharge^[^ ^14^ ^]^ or mechanical decay switch,^[^ ^15^ ^]^ they complicate device configuration and integration. Second, while numerous t‐TENGs have been reported, integration into fabric remains a challenge. For those that have been successfully integrated, they are often either separated into distinct parts or require complex fabrication procedures.^[^ ^13^, ^16^, ^17^ ^]^ In both scenarios, natural human emotions are sometimes hard to capture and track, as the design with separated components limits the location of integration on the fabric and the type of motion monitoring.

A new type of DC‐TENGs known as tribovoltaic nanogenerator (TVNG) has been invented.^[^ ^18^, ^19^, ^20^, ^21^, ^22^ ^]^ Eliminating the need for additional rectifiers or complex structure design, the simplest TVNG configuration consists of two components, a metal/semiconductor^[^ ^23^ ^]^ and a semiconductor.^[^ ^24^ ^]^ Such a combination exploits the physics of junction interfaces to provide self‐rectification of the current. Early studies have mostly focused on inorganic semiconductors such as Si and GaN, but their rigidity and brittleness hinder their long‐term and flexible applications.^[^ ^24^, ^25^ ^]^ Recently, flexible organic semiconductors have garnered increased attention in textile TVNG research, where representing examples are conductive polymers, primarily poly(3,4‐ethylenedioxythiophene): poly(styrenesulfonic acid) (PEDOT:PSS)^[^ ^18^ ^]^ or polypyrrole (PPy). Despite their great promise in textile electronics applications, there is still room for improvement in electrical properties, mechanical and chemical stability, and processability.^[^ ^26^ ^]^ One of the best approaches to achieve robustness and high electrical performance in e‐textiles is covalent modification of precursor textiles.^[^ ^27^ ^]^ Another valuable strategy is in situ formation of the functional compound on and within the fibers.^[^ ^28^, ^29^ ^]^ Good candidate classes for textile semiconductor research are MXenes^[^ ^30^ ^]^ and metal‐organic framework (MOFs)^[^ ^31^ ^]^ but remain mostly untapped in TVNG development.

In some of the textile‐based TVNGs that have been developed, some components remain rigid or separated from the textile, limiting garment integration.^[^ ^19^ ^]^ Although some have used metal foils^[^ ^32^ ^]^ or plastic films as substrates and integrated them into garments for monitoring physiological movements, the modulus mismatch between these devices and fabrics significantly affects the robustness and comfort of long‐term use.^[^ ^33^ ^]^ As an emerging conductive 2D MOF, copper‐benzenehexathiol (Cu‐BHT) has been recognized as a semiconductor material suitable for various electrical applications such as transistors and sensors.^[^ ^34^, ^35^ ^]^ Our previous studies have further demonstrated its potential in electrochemistry^[^ ^36^ ^]^ and energy harvesting.^[^ ^37^ ^]^

In the present study, we aimed to develop a fully textile TVNG solution that would be produced using a simple modification of common fabrics, seamlessly integrated into a garment, and used to generate a DC signal for physiological monitoring. We explored the in situ growth of MOF with semiconductor properties onto fabrics to realize the potential applications of TVNG. In our case, Cu‐BHT was directly grown on common cotton, serving as a novel p‐type semiconductor. Integrated with aluminum (Al) fabric, a Cu‐BHT‐based tribovoltaic textile (Cu‐BHT TVT) was developed, showcasing excellent stability and durability. Leveraging its full‐textile property, our flexible Cu‐BHT TVT can be seamlessly integrated into everyday clothing at different positions such as waistband and knee brace. Beyond powering electronics like calculators, these integrated Cu‐BHT TVTs can track daily motions (such as walking) and monitor body health (such as abdominal respiration).

## 2 Results and Discussion

**Figure 1:**
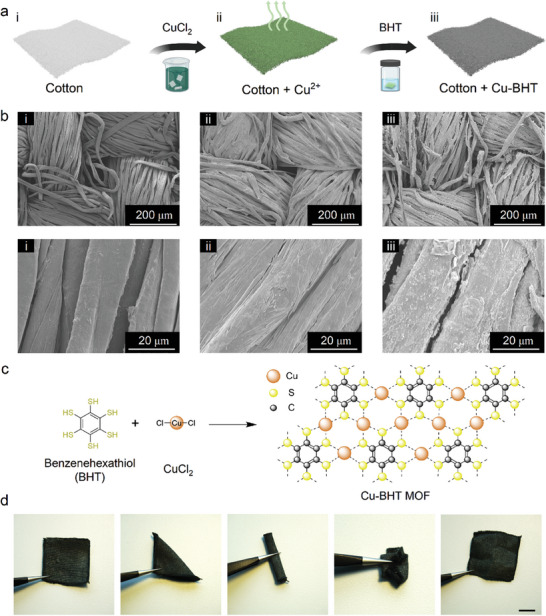
Fabrication and structure of the Cu‐BHT cotton. a) Schematic of the fabrication process diagram of Cu‐BHT cotton. b) SEM images showing different stages of cotton modification: pure cotton (i), cotton with CuCl_2_ bonding (ii), and cotton with Cu‐BHT growth (iii). c) Schematic of Cu‐BHT formation from BHT and copper (II) chloride. d) Photographs demonstrating the flexibility of cotton grown with Cu‐BHT under various external forces. Scale bar in (d): 1 cm.

**Figure** **1**a illustrates two crucial steps in the in situ growth of Cu‐BHT on textiles. One is the coordination of Cu ions (Cu^2+^) with cotton, and the other is the reaction between Cu^2+^ and BHT. When cotton is immersed in a Cu^2+^‐rich solution, coordination bonds are generated between Cu^2+^ and hydroxyl groups on cellulose chains,^[^ ^38^ ^]^ resulting in a stable Cu^2+^‐cellulose complex and thus strong attachment of Cu^2+^ to the cotton surface. Compared to the pristine cotton that features relatively clean and smooth fibers on the micro‐scale, as evidenced by SEM imaging (Figure 1b(i)), the surface of Cu^2+^‐treated cotton fibers is visibly decorated with copper chloride crystals (Figure 1b(ii)). It can be seen that the cotton modified in this way retains its fine fibrous structure, with only the surface being affected. After the modification process, the fabric attains a darker grey shade, and the presence of Cu‐BHT MOF can be easily verified by measuring the electrical conductivity of the sample after drying (181.8 S cm^−1^). Importantly, no obvious change was observed after hand rubbing the Cu^2+^‐treated cotton (Figure S1, Supporting Information), demonstrating the stability of Cu^2+^ on textiles. As soon as the Cu^2+^‐rich cotton is soaked in a BHT‐containing solution, Cu^2+^ reacts with BHT, forming a Cu‐BHT network via Cu─S bonds (Figure 1c). Finally, this process yields a flexible and durable Cu‐BHT cotton that can withstand folding, rubbing, and twisting (Figure 1d).

As depicted in Figure S2 (Supporting Information), while the Cu‐BHT is characterized by the well‐distinguishable crystalline peaks^[^ ^37^ ^]^ at 11.8°(100 plane), 26.6°(001 plane), 35°(201 plane), the XRD patterns of pristine cotton and the Cu‐BHT cotton are almost undistinguishable, with no obvious characteristic peaks of Cu‐BHT being observed in Cu‐BHT sample. This is mainly because the cotton is dominating the XRD pattern with the characteristic cellulose Iβ diffraction peaks.^[^ ^39^ ^]^ This is, however, quite expected, as the amount of MOF grown on the textile is small compared to the sheer amount of cellulosic fibers present in the material (see also SEM images, Figure S3, Supporting Information). Despite being sufficient to both conduct electricity and act as a semiconductor in the TVT, the Cu‐BHT grown in the cotton substrate is undetectable by XRD.

**Figure 2:**
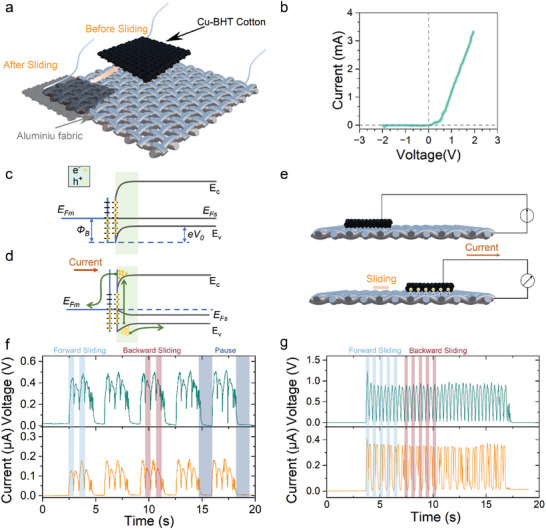
Working principle and output of Cu‐BHT TVTs. a) 3D schematic diagram of Cu‐BHT TVT, which consists of Cu‐BHT cotton and Al fabric. b) Current‐Voltage curves at the Cu‐BHT/Al interface. A light blue background shows the original data; a black solid line represents fitted data. Energy band diagram at the Cu‐BHT/ Al interface in (c) contact and (d) sliding states. e) Schematic illustrating electricity generation in the Cu‐BHT TVT. f, g) Typical voltage and current output of a Cu‐BHT TVT with a slider area of 9 cm^2^.

**Figure** **2**a illustrates the construction and operation of the Cu‐BHT TVT device. In our case, the device only consists of Cu‐BHT‐modified cotton and Al fabric. Cu‐BHT is an ambipolar semiconductor;^[^ ^34^ ^]^ when it contacts a metal with a lower work function, it behaves as a p‐type semiconductor with holes as the main charge carriers. In our case, the work function of Al (4.28 eV)^[^ ^19^ ^]^ is lower than that of Cu‐BHT (4.94 eV),^[^ ^40^ ^]^ which should theoretically provide a Schottky junction required for the TVNG operation. To verify the formation of such a junction, we have measured the current–voltage (*I–V*) characteristics of the Al fabric/Cu‐BHT cotton junction. When a forward bias is applied, current flows from the cotton to the Al, and the device displays a conductive state. As presented in Figure 2b, the *I–V* curve demonstrates a typical Schottky junction behavior due to the contact of semiconductor Cu‐BHT modified cotton and metal Al fabric.

Explained simply: before the contact, the electrons in Al had higher energy levels than those in Cu‐BHT. Upon contact, these high‐energy electrons of the Al metal will diffuse into the Cu‐BHT, filling lower empty energy levels. This results in the Al side becoming positively charged and the Cu‐BHT side being negatively charged, creating a built‐in electric field pointing from Al to Cu‐BHT. Equilibrium is achieved when the diffusion of electrons from the Al to Cu‐BHT balances out the built‐in electric field. The Fermi level aligns and the Cu‐BHT energy band bends upward as shown in Figure 2c. In operation as a TVT device, during the sliding of the materials forming the junction, electron‐hole pairs generate and recombine in dynamic balance. The built‐in electric field separates and directs formed electron‐hole pairs, preventing recombination and facilitating external current flow as shown in Figure 2d.

In practical applications, human body movements are typically irregular and discontinuous. Therefore, during the device output testing, incorporating pauses during forward‐backward motions better simulates real‐world scenarios. The testing model setup is illustrated in Figure S4 (Supporting Information), with stable output voltage and current demonstrated in both Figures 2f, g. Whether sliding is continuous or discontinuous, the motion is in an forward and backward circular fashion. During both sliding motions, Cu‐BHT TVT outputs self‐rectified DC signals. In the test presented in Figure 2f, a pause occurs after every two cycles of movement, with each independent forward or backward movement generating a positive DC/voltage output. On the other hand, Figure 2g shows continuous sliding without pauses (changing directions at 1.5 Hz, 4N; setup shown in Figure S5, Supporting Information), simulating continuous activities like running or walking. However, the amplitude of output signals from these tests varies significantly, highlighting that the device performance is highly influenced by multiple factors like sliding frequency and velocity.

**Figure 3:**
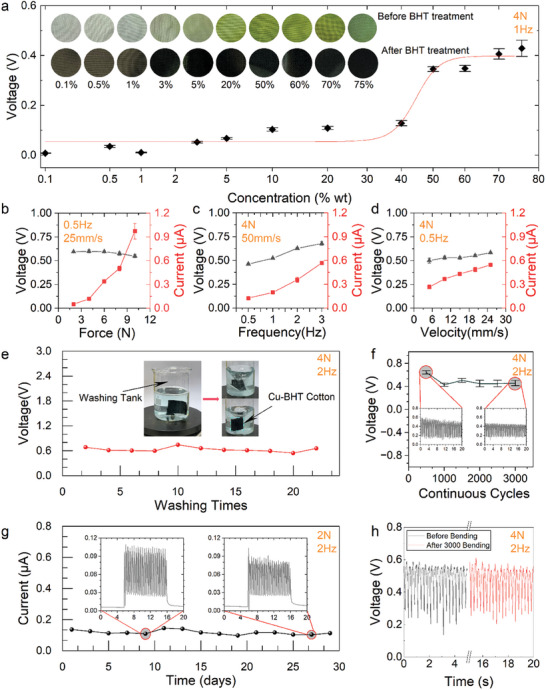
Effect of chemical and physical parameters on the output performance of Cu‐BHT TVTs. a) Effect of Cu^2+^ concentration on the output voltage. Insets show corresponding photographs of cotton samples before and after the Cu‐BHT reaction. Output of voltage and current with different sliding parameters such as (b) force, c) frequency, and d) velocity. e) Voltage output of Cu‐BHT TVT after 20 washes. Insets display photographs before and during washing. f) The voltage output of Cu‐BHT TVT after 3000 continuous sliding cycles. g) Current output of Cu‐BHT TVT after 30 days of storage. h) The voltage output of Cu‐BHT TVT is in its original state after 3000 bending cycles.

The output performance of the Cu‐BHT TVT device depends not only on the parameters of the fabrication process but also on the testing conditions. For example, when cotton is soaked in a BHT‐based solution, each BHT molecule reacts with multiple Cu ions, with the reaction direction sustained by a sufficient supply of Cu ions. While the concentration of the BHT solution remains constant, the impact of varying CuCl_2_ solution concentrations on the device output is explored. Higher densities of Cu ions on the cotton surface increase the likelihood of BHT interacting with these ions, resulting in a denser and thicker Cu‐BHT film network. This observation is depicted in the inset of **Figure** **3**a, where the color of Cu‐BHT cotton darkens with higher Cu ion concentrations, correlating with increased conductivity (Table S1, Supporting Information). Concurrently, the output voltage (Figure 3a) also rises (Figure S6, Supporting Information), as a more complete Cu‐BHT film on the cotton surface creates a larger interface during friction with the Al fabric. However, once the concentration of Cu ions reaches a certain level, further changes in output are minimal, which is mainly limited by the available BHT molecules in the solution. In our case, 70 wt.% of CuCl_2_ was selected as the final concentration for further investigation.

Besides the preparation process, mechanical parameters during the sliding process significantly affect the final output signal of the Cu‐BHT TVT. Thus, we studied motion parameters such as sliding force, frequency, and relative sliding velocity in this work. For instance, as the friction force rose from 2 to 10 N, the average current increased from 0.05 to 0.97 µA, while the average voltage remained stable at 0.6 V (Figure 3b). Higher sliding forces lead to closer contact at the interface, generating more electron‐hole charges and thereby increasing current density.^[^ ^19^ ^]^ On the other hand, the external load voltage decreases slightly, and the current increases due to the decrease in internal resistance under higher load pressure^[^ ^41^ ^]^(see detailed explanation in Note S1, Supporting Information). While the voltage is mainly influenced by the barrier height among materials and surface conditions. During the sliding process, the Fermi level of the Cu‐BHT decreases slightly toward the valence band and thus to a lower energy level, resulting in an insensitive output voltage.^[^ ^19^, ^22^ ^]^ Similarly, increasing sliding frequency and velocity raised the output current (Figures S7–S9, Supporting Information), while voltage showed insensitivity to these parameters. Higher velocity and frequency dissipate more friction energy, exciting additional electron‐hole pairs at the material interface and boosting current output.

In everyday use, the longevity of clothing is predominantly influenced by washing, folding, and bending, with storage conditions also impacting fabric integrity. On this basis, we conducted 20 washing cycles to test the durability of the developed Cu‐BHT TVT. Figure 3e shows negligible fabric debris post‐wash. Concurrently, the TVT device exhibited consistent output signal amplitudes, demonstrating robust washability. As illustrated in Figure S10 (Supporting Information), the contact angle of the Cu‐BHT cotton is 137°. Following a stay period of 5 min or more, the contact angle remains at 130°. The hydrophobic properties of the Cu‐BHT cotton are most likely due to the Cu‐BHT being mostly comprised of aromatic rings and its hydrophilic thiols being occupied by the coordination of Cu^2+^.

Long‐term durability assessments of Cu‐BHT TVT (Figure 3f, subjected to 4 N force at 2 Hz) and 30‐day storage (Figure 3g) were conducted separately. Initially, in Figure 3f, the output voltage slightly decreased from 0.6 V to a stable 0.45 V due to variations in Cu‐BHT material height on the cotton surface. During friction testing, the applied stress tends to concentrate on the higher protrusions. Following an initial period of friction, any unstable Cu‐BHT layers detached, leaving the remainder securely attached to the cotton surface. As illustrated in Figure S3 (Supporting Information), there are no discernible changes to the surface of the Cu‐BHT cotton sample before and after the cyclic friction tests (10,000 cycles). This indicates that the growth of the Cu‐BHT on the textile surface provides good adhesion of the MOF to the fibers, which in turn ensures stable output performance of the developed Cu‐BHT TVT even after an extended friction test. Moreover, either after 30 days or 3 months of storage, the output current remains stable at ∼0.1 µA as shown in Figure 3g and Figure S11 (Supporting Information).

Subsequently, the textile underwent 3000 bending cycles using a linear Instron motor, detailed in Figure S12 (Supporting Information). Post‐bending, the voltage output of the TVT remained stable, as depicted in Figure 3h. This stability underscores the cotton's excellent flexibility, affirming that the Cu‐BHT adhered to the cotton surface maintains robust stability. As shown in Figure S13 (Supporting Information), an increase in humidity from 38% to 70% is accompanied by a reduction in output voltage from 0.55 to 0.45 V. This is attributed to a decline in charge generation at the interface.^[^ ^23^ ^]^ To explore more possibilities with fabric substrates, we have included three other fabrics‐including knitted 100% cotton (different structure), 97% cotton + 3% Lycra‐blend, and linen. As illustrated in Figure S14 (Supporting Information), Cu‐BHT has been well‐grown on all of these fabrics, resulting in similar output values obtained from their corresponding devices.

**Figure 4:**
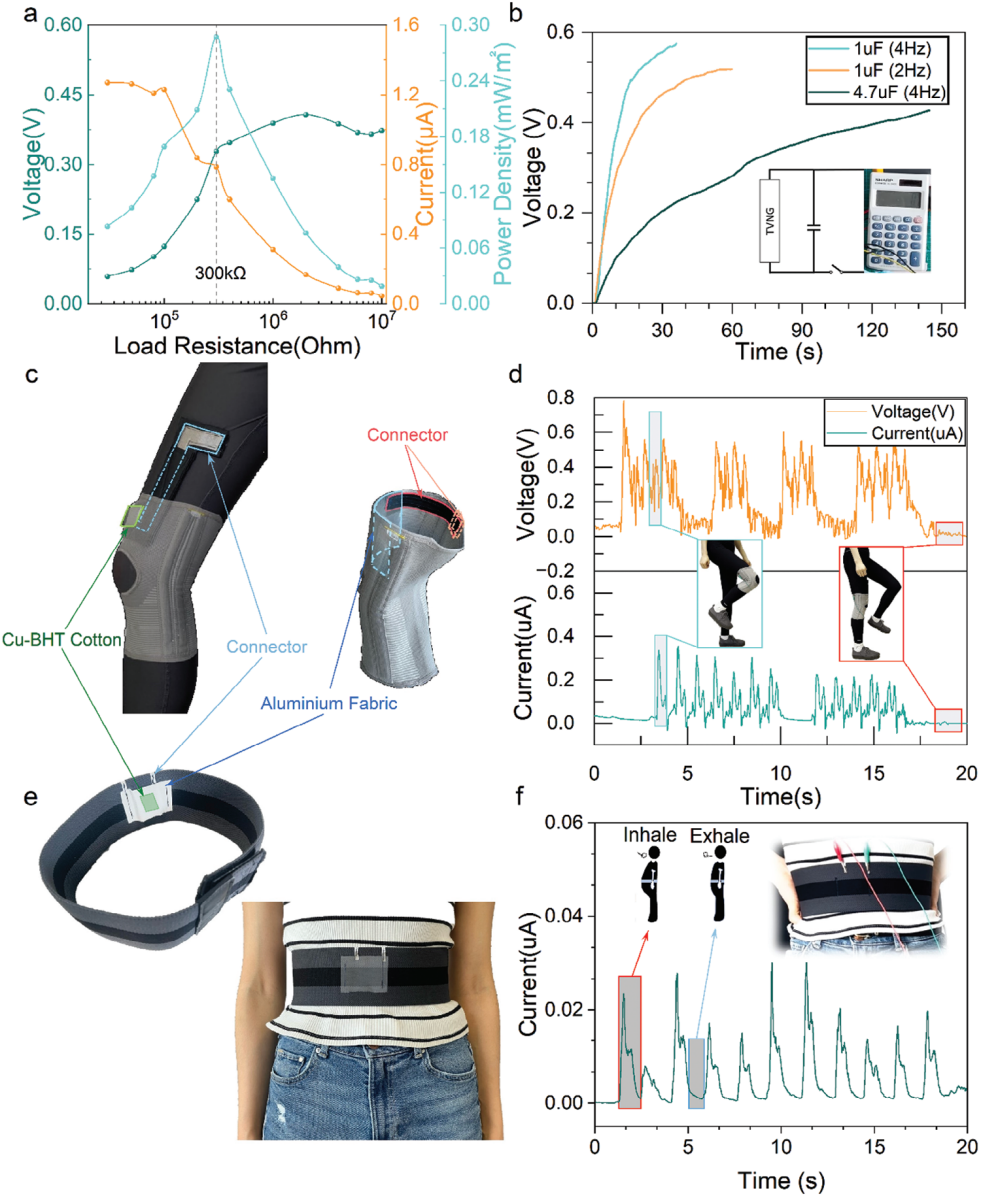
Powering and sensing applications of Cu‐BHT TVTs. a) Variation of output current, voltage, and power density of Cu‐BHT TVT with different external load resistances. b) Voltage profiles of two capacitors charged by a single Cu‐BHT TVT in different sliding frequencies. A digital calculator (inset) was powered by six Cu‐BHT TVT connected in series. Photographs of Cu‐BHT TVT‐based wearable (c) kneepad and (e) belt. d) Voltage and current monitoring signals for the walking process. f) Current signals during abdominal breathing.

Power density stands as a critical metric for energy harvesters. In our case, the output current remained nearly constant at load resistances <10 kΩ, declining with higher resistances. **Figure** **4**a illustrates achieving a maximum output power density of 0.287 mW m^−2^ at 300 kΩ load resistance, showcasing the promising potential for the TVT device as a wearable DC power supplier. Xu's research highlights substrate hardness influencing device output; stainless steel yielded optimal results.^[^ ^42^ ^]^ In other related research, metal is typically electroless plating^[^ ^19^ ^]^ onto fabric to serve as the TVNG component. However, this technique often compromises the fabric's original flexibility. In contrast, our Cu‐BHT TVT integrates directly onto cotton, preserving fabric flexibility, and facilitating seamless electronic integration.

Moreover, capacitor charging tests revealed efficient performance: a 1 µF capacitor charged to ∼0.5 V in 60 s at 2 Hz and ∼0.55 V in 30 s at 4 Hz, aligning with frequency‐dependent output trends. As sliding frequency increases, there is a slight increase in voltage output, while the output current shows significant improvement. For a 1 µF capacitor, high current density results in a shorter charge time; when charging the capacitor at a higher sliding frequency, the charging time is reduced due to increased current density. For a 4.7 µF capacitor, it takes 150 s to charge to ∼0.4 V at 4 Hz. By connecting multiple TVT devices in series, different device voltage requirements can be met. In this experiment, six TVT devices were successfully connected in series to charge a 1 µF capacitor. Upon reaching 1.6 V, the capacitor was connected to a calculator (Movie S1, Supporting Information), causing the calculator's screen to briefly illuminate, as depicted in Figure 4b (insert figure).

Given its structural flexibility and portability, the developed TVT holds the potential for integration into wearable accessories for practical applications. In this study, we incorporated the Cu‐BHT TVT into wearable items such as a kneepad (Figure 4c) and belt (Figure 4e) for monitoring lower limb movements (Figure 4d) and abdominal aspirations (Figure 4f). During activities like walking and jumping, friction between the Cu‐BHT cotton and Al fabric at the knee joint generates output signals, enabling tracking of knee motion, as shown in Figure 4d. The TVT device at the knee joint experiences both frictional and contact‐separation motions, resulting in a mixed output signal. Future applications could enhance output signals by connecting multiple devices in series and increasing contact areas to power more devices. Besides joint movements, the TVT can detect subtle movements such as aspiration. Cu‐BHT cotton and Al fabric were affixed to the inner surface of the belt in our case. During abdominal breathing, relative movement and friction between the two fabrics produce DC signals, reflecting respiratory rate, as depicted in Figure 4f and Movie S2 (Supporting Information). This capability opens possibilities for future respiratory monitoring scenarios using TVT devices.

## 3 Conclusion

In this work, we developed a flexible TVT featuring a simple two‐layer structure comprising Al fabric as the metal component and Cu‐BHT cotton as the semiconductor. The Cu‐BHT TVT exhibited stable voltage and current outputs under both continuous and discontinuous sliding cycles. Through optimization of physical and chemical parameters, the developed TVT achieved high performance with outputs of ∼0.9 V in voltage and ∼1 µA in current. Extended durability tests including 20 wash cycles and 3 months of storage showed consistent performance comparable to its initial state. Even after enduring thousands of frictions and bending cycles, the Cu‐BHT TVT maintained excellent flexibility and output stability. Moreover, the successful integration of the Cu‐BHT TVT into wearable devices enabled continuous monitoring of human motion and respiratory signals. This advancement not only showcases the potential of TVTs to enhance wearable technology functionality but also lays the groundwork for their application in comprehensive health monitoring and personalized medical devices.

## Conflict of Interest

The authors declare no conflict of interest.

## Author Contributions

Y.W., Y.L., and C.M. conceived the project and designed the experiments. Y.L. fabricated the TVT samples and devices. Y.L., A.S., and Y.W. ran the experiments and analyzed the data. Y.L. and Y.W. wrote the initial draft of the manuscript. Y.W., A.S., and C.M. reviewed the manuscript. Y.W. and C.M. supervised the project.

## Supporting information



Supporting Information

Supplemental Movie 1

Supplemental Movie 2

## Data Availability

The data that support the findings of this study are available from the corresponding author upon reasonable request.
