# Supplementary material for: Metal‐Organic Framework‐Based Tribovoltaic Textile for Human Body Signal Monitoring
Source: Adv Sci (Weinh). 2025 Feb 5;12(17):2414086. doi: 10.1002/advs.202414086 (PMC12061281; doi:10.1002/advs.202414086)
Supplement: Supplementary file 1 — Supporting Information [file ADVS-12-2414086-s002.docx]

Supporting Information

Metal–Organic Framework Based Tribovoltaic Textile for Human Body Signal Monitoring

Yuanlong Li^1^, Yinghong Wu^1, 2, *^, Alexander V Shokurov^1^, Carlo Menon^1, *^

1. Biomedical and Mobile Health Technology Laboratory, Department of Health Sciences and Technology, ETH Zurich, Lengghalde 5, 8008 Zürich, Switzerland

2. National Engineering Research Center of Green Recycling for Strategic Metal Resources, Institute of Process Engineering, Chinese Academy of Sciences, Beijing 100190, China

*Corresponding Authors: [yhwu@ipe.ac.cn](mailto:yhwu@ipe.ca.cn) (Y. Wu) and [carlo.menon@hest.ethz.ch](mailto:carlo.menon@hest.ethz.ch) (C. Menon)

**Experimental Section/Methods:**

*Materials*: Copper chloride (CuCl_2_), chlorobenzene (CB) and Ethanol (EtOH) used in this study were purchased from Sigma-Aldrich and used as received without further purification. 1,2,3,4,5,6-benzenehexathiol (BHT) was bought from BLD Pharmatech. Aluminum fabric (EGLA200 AL) were purchased from Texfire Technical Fabrics (Spain) and used as received.

*Synthesis of Cu-BHT*: BHT was first dissolved in CB to form a 0.5 mM BHT solution. CuCl_2_ was dissolved in deionized water (DI water) to yield different concentration Cu ions solution. Pure cotton samples were pretreated by soaking in a 10 wt% NaOH solution overnight for desizing and cleaning, followed by washing with DI water and soaking in the prepared Cu ions solution for 4 hours. Subsequently, Cu-cotton was air-dried in a fume hood. The BHT solution was then applied to cover Cu cotton samples and let react for 30 minutes, followed by washing with EtOH and DI water, resulting in the formation of a Cu-BHT film observable on the cotton surface.

*Device fabrication and integration*: Devices for motion tracking: Al fabric was affixed to the interior of kneepads, while Cu-BHT cotton was sewn onto pants with conductive fabric used as traces for both Al fabric and Cu-BHT cotton.

Devices for respiration monitoring: Cu-BHT cotton, backed with cushioning sponge, was initially sewn onto a belt. Subsequently, Al fabric was sewn atop the cotton, with conductive fabric used for wiring the Al fabric and Cu-BHT cotton.

*Characterization*: The morphologies of various samples were examined using a scanning electron microscope (SU5000, Hitachi). The phase of the samples was identified using an X-ray diffractometer (XRD machine PANalytical X'PERT Pro). Current-voltage characteristics were measured using a potentiostat (μStat-i400, MetrOhm DropSens) in a two-electrode configuration. Samples were connected to the potentiostat using flat-nosed alligator clips with Cu-BHT cotton connected to the working electrode and Al fabric connect to the ground electrode, and voltage was swept from -2V to +2V while measuring current. Samples’ resistances were measured using a multimeter (Model 177, FLUKE). The mechanical tests were conducted using an electric dynamic test instrument (Electropuls E3000, Instron). The output of the Cu-BHT TVT was evaluated using an oscilloscope (MDO34, Tektronix) and a low-noise current preamplifier (SR570, Stanford Research Systems).

The washing test was conducted by immersing Cu-BHT cotton samples in a tank containing 100 mL of DI water and 1 mL of laundry detergent, stirred magnetically at 30 °C for 30 minutes. Post-washing, samples were air-dried at room temperature. Wetting angle studies for the developed textiles were carried out using a Biolin Scientific Theta Lite optical tensiometer in sessile drop mode. Distilled water was used as a testing liquid, initial droplet volume was 5 µL. The observed contact angles are reported without correction for the substrate roughness. Sensirion Digital humidity and temperature sensor was used to detect humidity. Humidity of 38% is based on the local environment. By means of a continuous spray of water mist in a closed chamber, a humidity of 70% is achieved.


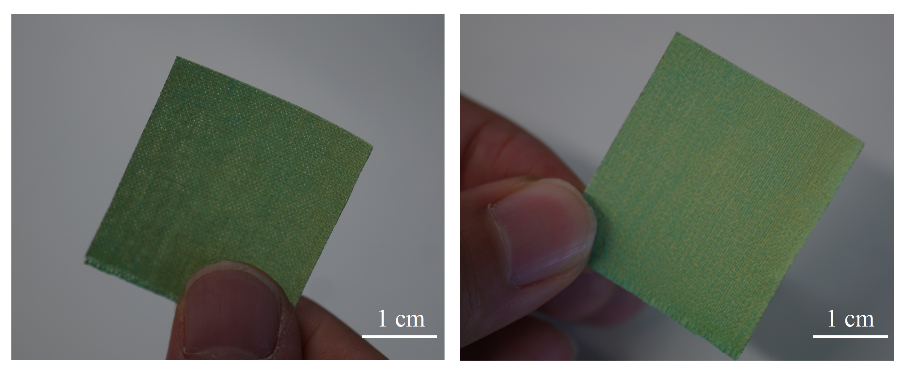


**Supplementary Fig.S1 | Photographs of Cu-rich cotton before and after rubbing.** After immersion in the CuCl_2_ solution, the cotton surface exhibits green and blue hues. The CuCl_2_ crystals remain firmly attached after several rubs with no noticeable detachment.


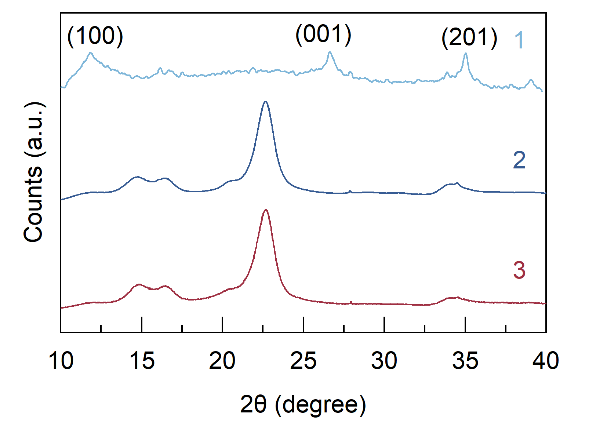


**Supplementary Fig.S2 | XRD pattern of different samples.** (1. Cu-BHT powders; 2. Cu-BHT cotton; 3. Pristine cotton)


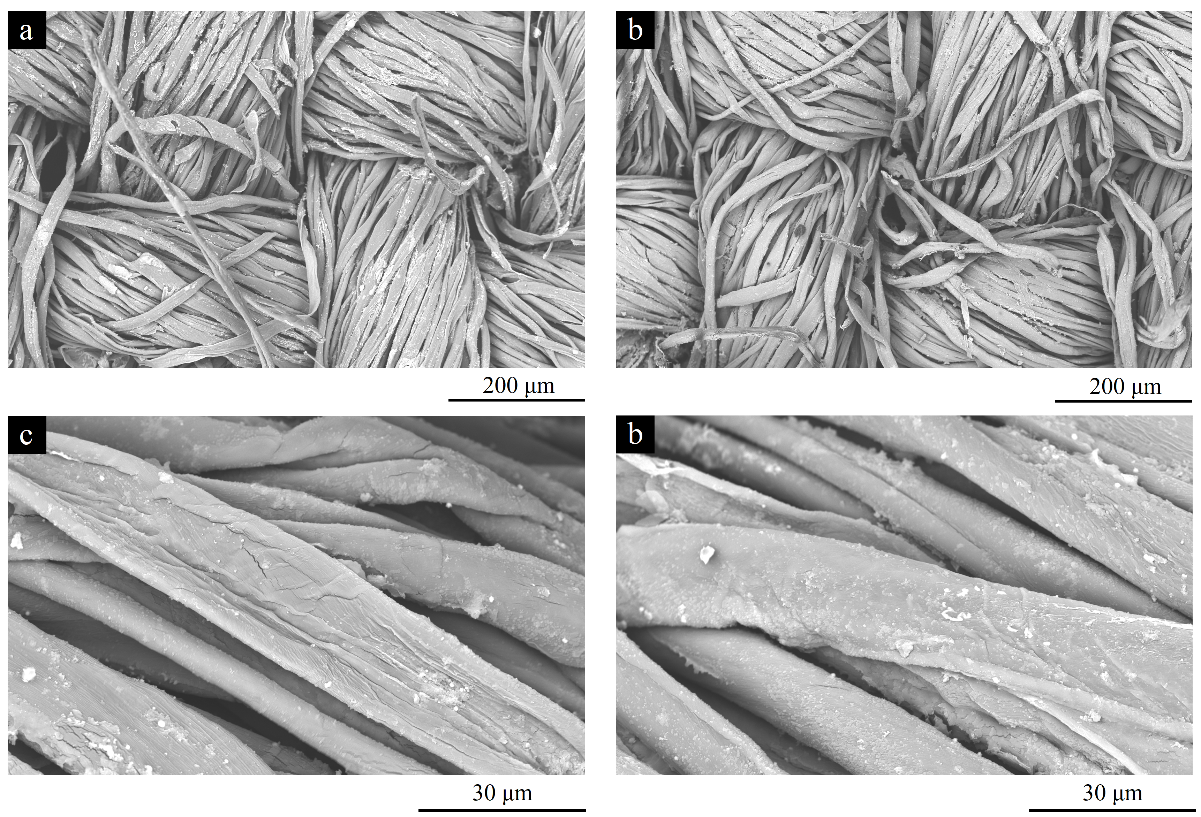


**Supplementary Fig.S3 Surface wear conditions before and after cyclic testing.** (a) and (c) Surface conditions before cyclic friction test. (b) and (d) Surface conditions after cyclic friction test at various magnifications.

**
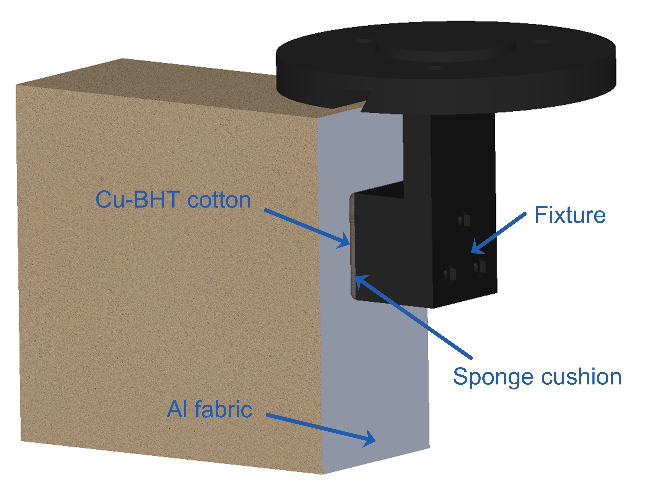
**

**Supplementary Fig.S4 | Schematic diagram of the Cu-BHT TVT setup for mechanical and electrical tests using an electric dynamic test instrument.** A fixture prepared via 3D printing is affixed onto the test instrument. The Cu-BHT TVT is secured to the fixture with a sponge cushion layer. Parameters such as frequency, velocity, and displacement are controlled through the program of the test instrument.


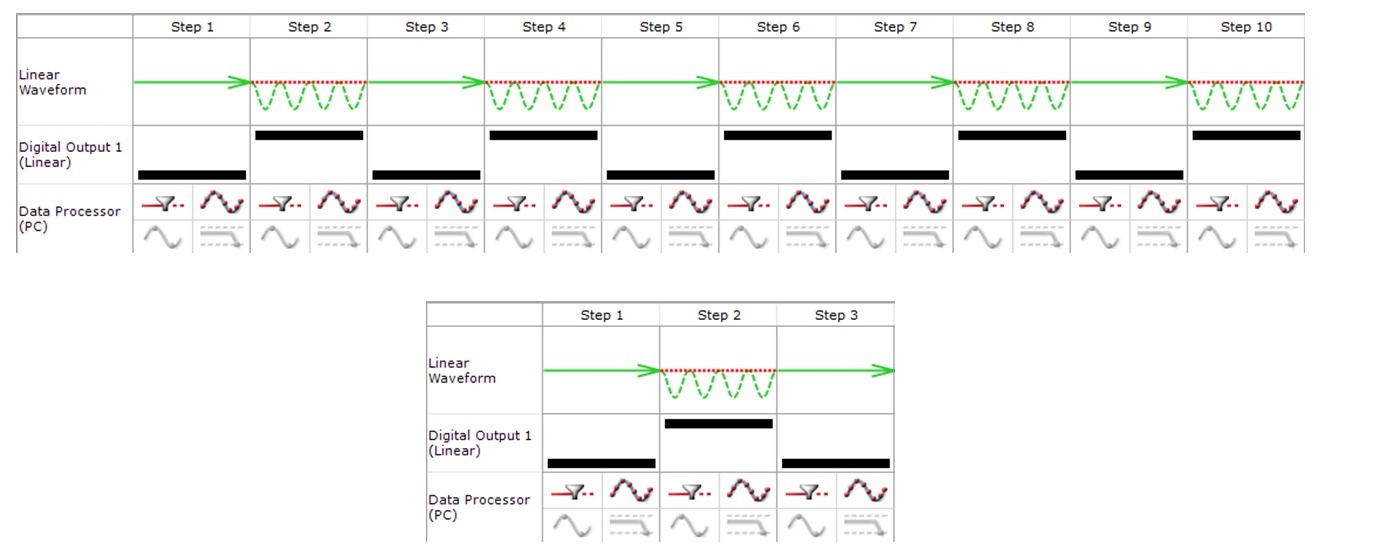
**Supplementary Fig.S5 | Program setups for discontinuous and continuous cycles on the electric dynamic test instrument.** Method 1 includes 5 cycles, each comprising 2 movements with 2 seconds of rest between cycles. Method 2 involves continuous movement without interruption.


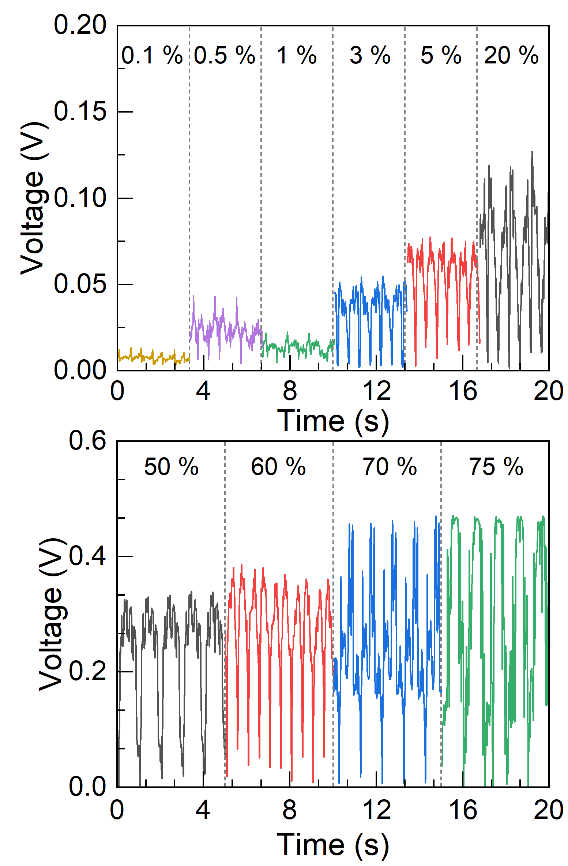
**Supplementary Fig.S6** | **Effect of CuCl_2_ concentration (0.1-70%) on device output voltage.** Increased CuCl_2_ concentration correlates with higher output voltage.

**
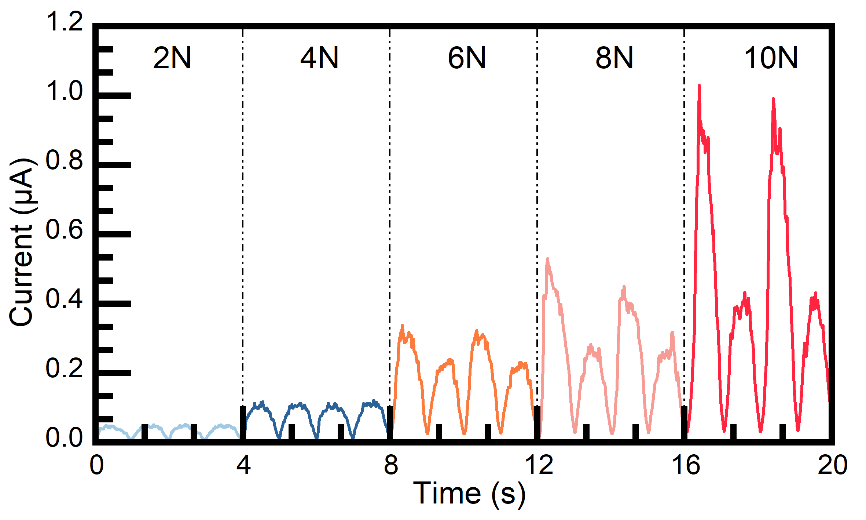
**

**
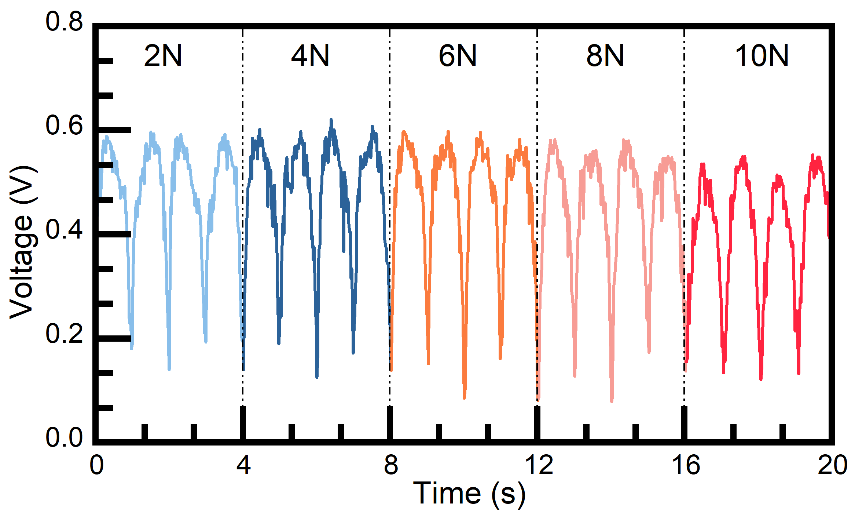
**

**Supplementary Fig.S7** | **Effect of applied force (2-10 N) on device output.** Increasing frictional resistance shows no significant impact on output voltage but significantly increases output current.

**
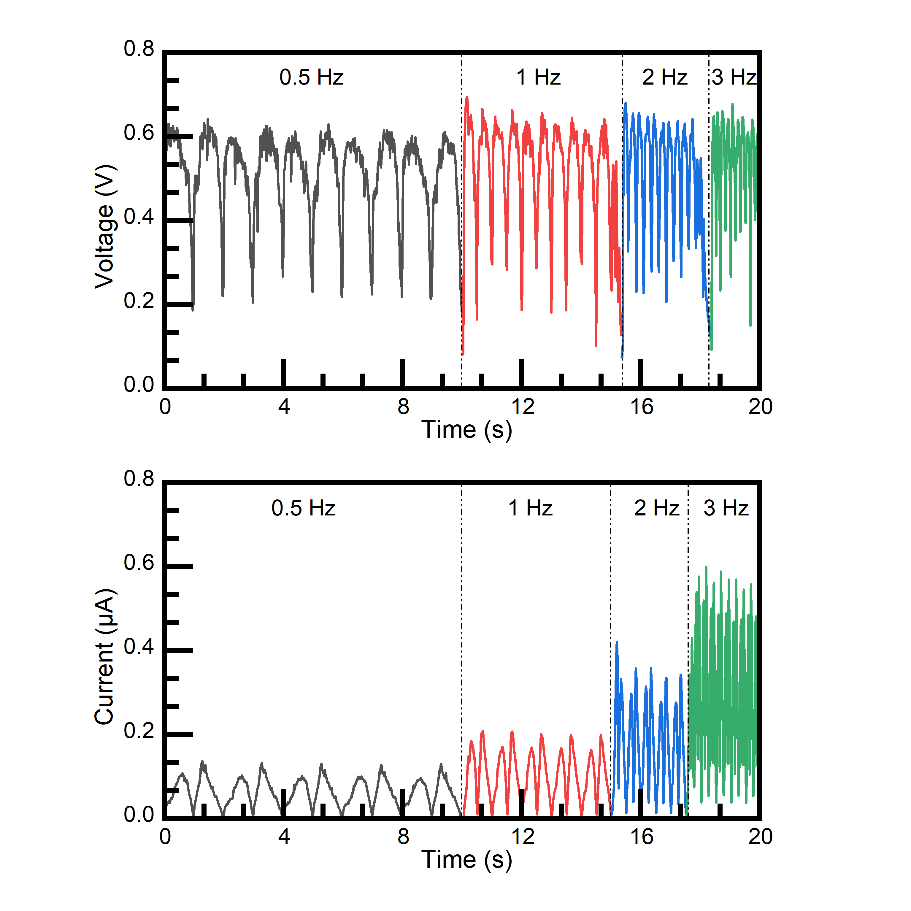
Supplementary Fig.S8 | Effect of applied frequency (0.5-3 Hz) on device output.** Increasing frequency does not significantly affect output voltage but significantly increases output current.

**
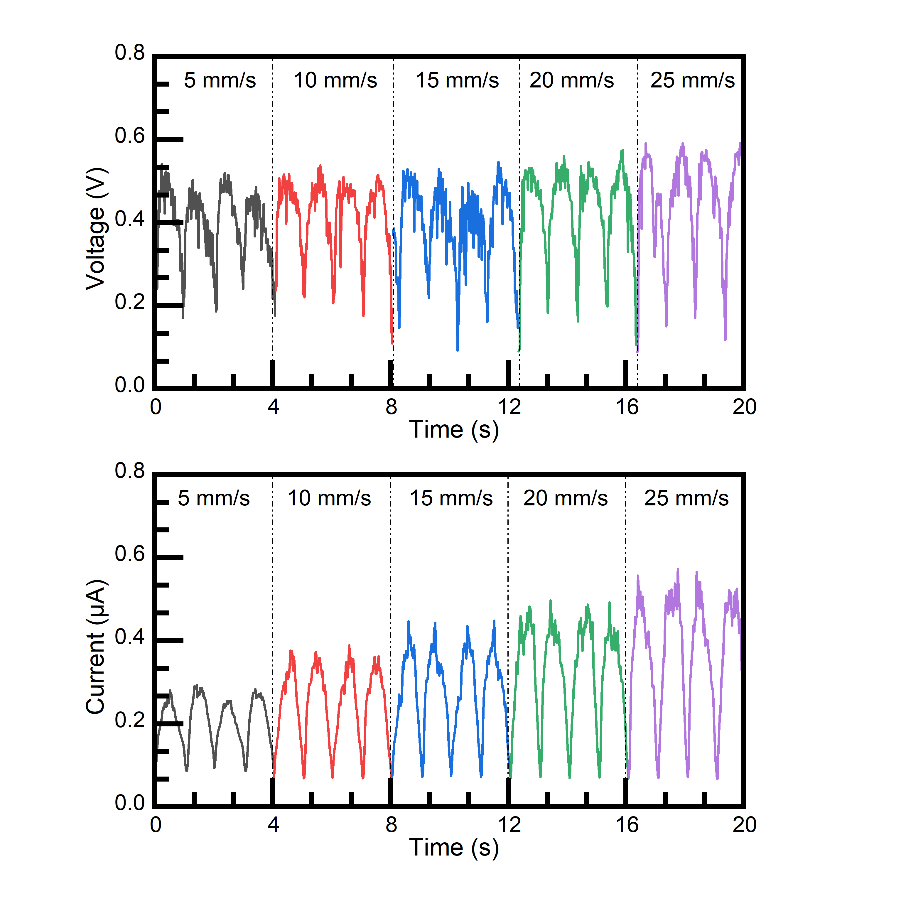
**

**Supplementary Fig.S9 | Effect of applied velocity (5-25 mm/s) on device output.** Increasing velocity shows no significant impact on output voltage but increases output current.


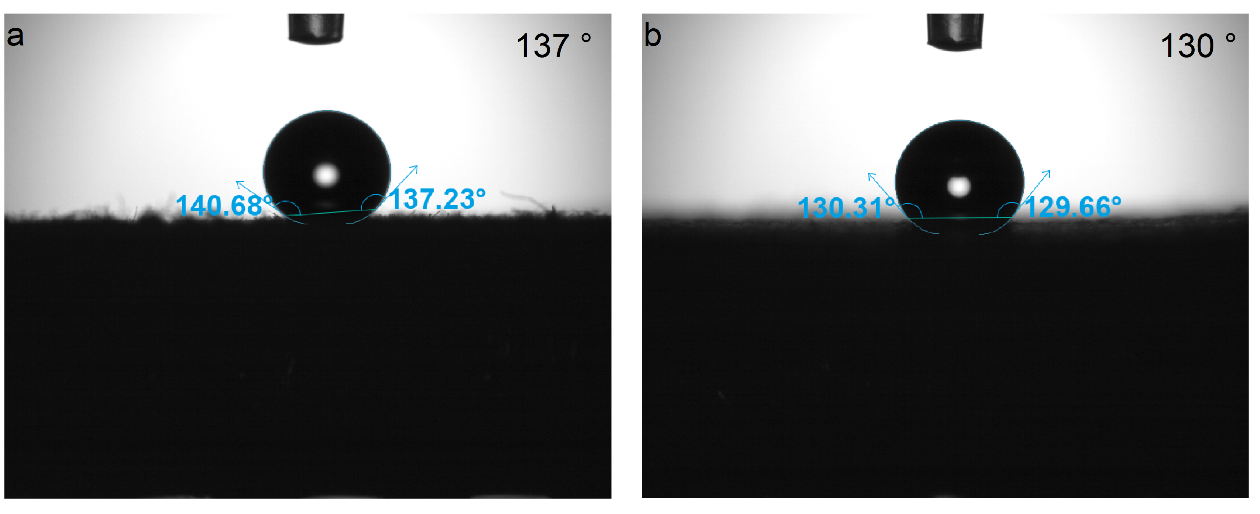


**Supplementary Fig.S10 | Water contact angles of Cu-BHT cotton samples.** a) upon deposition of the droplet and b) after five-minute stay of the water drop on the same spot of the sample.

**
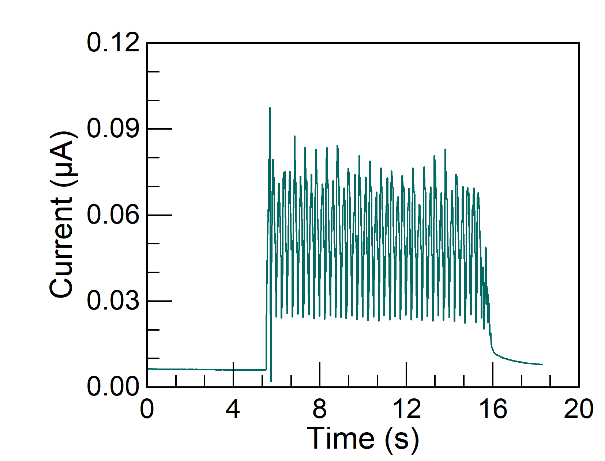
Supplementary Fig.S11 |. Device output after 3months of storage.** The obtained current remains similar to its initial state.

**
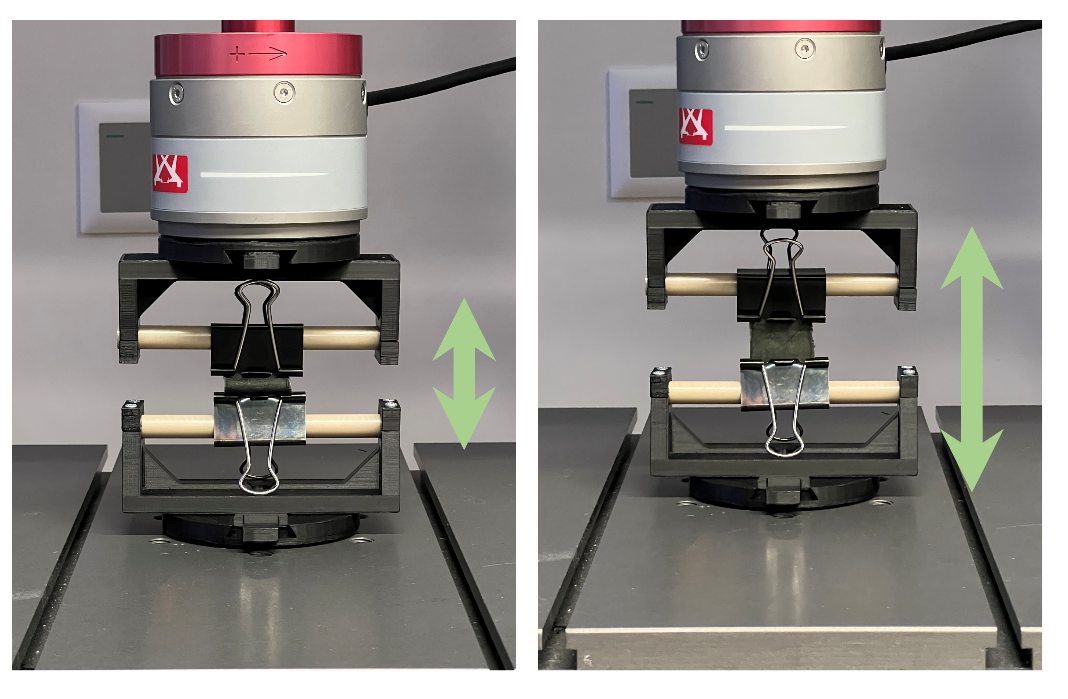
Supplementary Fig.S12 | Photographs of Cu-BHT cotton in bending and releasing states.** Cotton is fixed on a fixture and manipulated with the Instron for up-and-down movements to achieve bending.


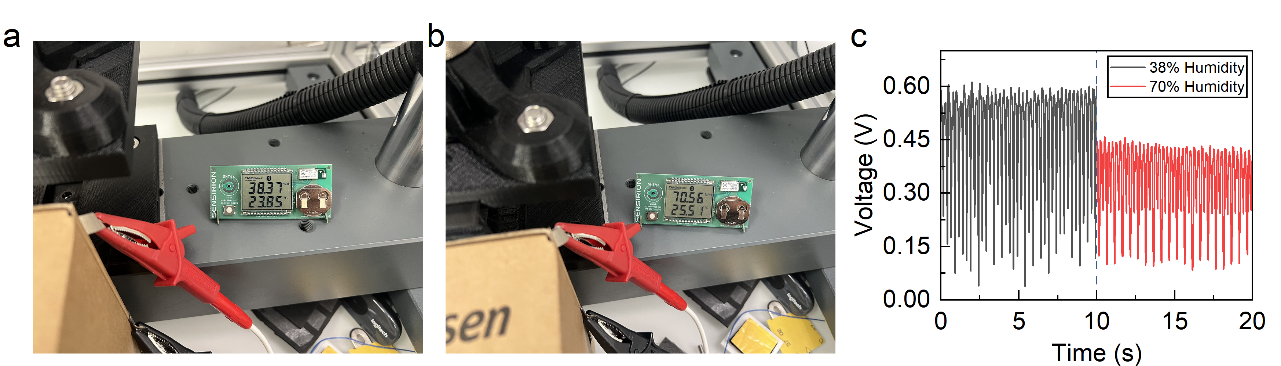


**Supplementary Fig.S13 | Effect of different humidity on the output performance.** (a) Test under general 38% room humidity. (b) Test under 70% humidity. (c) Output performance under different humidity environment.


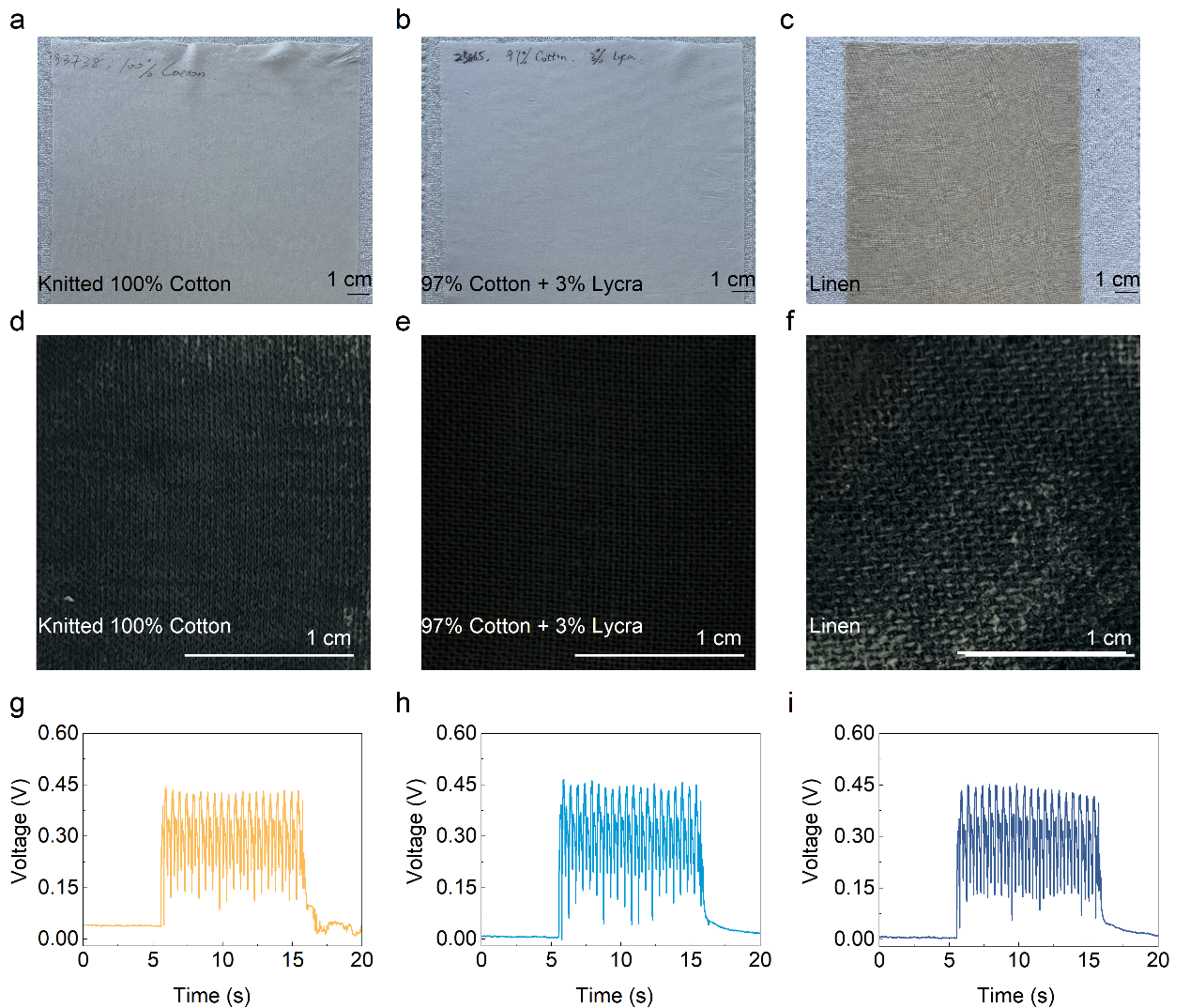


**Supplementary Fig.S14 | Effect of different fabric substrates on the output performance of the studied TVT.** 100% knitted cotton in (a) pristine state and (d) after Cu-BHT growth. 97% cotton + 3% Lycra blend (b) before and (e) after Cu-BHT growth on it. Linen fabric before (c) and (f) after Cu-BHT growth. Output voltage of (g) knitted 100% cotton, (h) 97% cotton + 3% Lycra blend, and (i) linen Cu-BHT based TVT devices.

**Supplementary Table S1**: Effect of CuCl_2_ concentration on the colour and resistance of Cu-rich cotton.


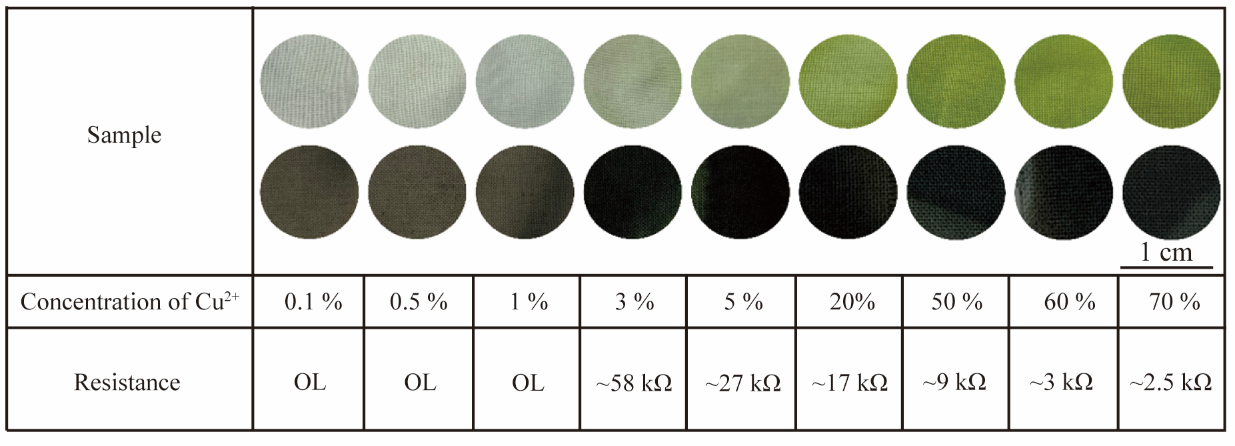


Annotation: Overload (OL).


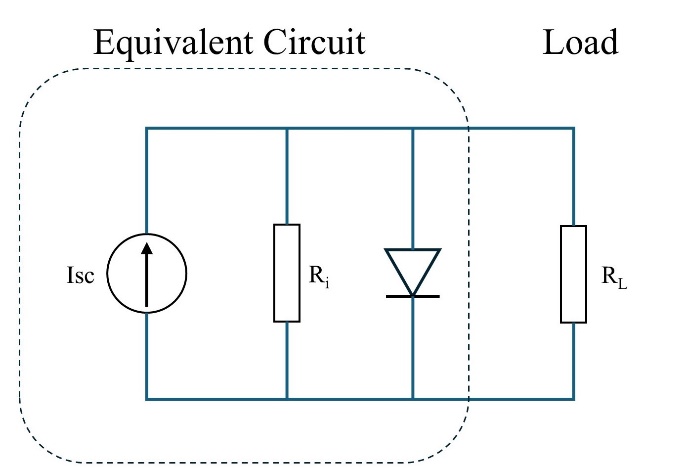
**Supplementary Note 1: Effect of friction force on the output voltage and current of Cu-BHT TVT.** Cu-BHT TVT can be regarded as a current source^[1]^ with an internal resistance (Ri) and connected in parallel with a resistor (RL), as illustrated in the following figure:

**Supplementary Fig.S15 | The equivalent circuit of Cu-BHT TVNG.**

The application of a larger force results in an increase in interfacial contact between Al and Cu-BHT, which in turn leads to a reduction in Ri. This results in a reduction of Ri || RL and the voltage at the external load.

On the other hand, an increase in pressure leads to a closer contact between two textile components, and thus an increase in generated charges at the interface, which results in an increase in both voltage and current. At the same time, such intimate contact will also lead to decrease of the internal impedance of the junction^[2]^, further leading to the enhanced current. Therefore, for the voltage, two opposing factors balance each other to achieve a slight decrease. In contrast, for the current, two positive factors lead to a significant increase.

Reference:

[1] X. Guo, J. You, D. Wei, J. Shao, Z. L. Wang, Applied Physics Reviews 2024, 11, 021415.

[2] Z. You, S. Wang, Z. Li, Y. Zou, T. Lu, F. Wang, B. Hu, X. Wang, L. Li, W. Fang, Y. Liu, *Nano Energy* **2022**, *91*, 106667.
